# Prognostic implications of pericardial and pleural effusion in patients with cardiac amyloidosis

**DOI:** 10.1007/s00392-020-01698-7

**Published:** 2020-09-10

**Authors:** Christina Binder, Franz Duca, Thomas Binder, René Rettl, Theresa Marie Dachs, Benjamin Seirer, Luciana Camuz Ligios, Fabian Dusik, Christophe Capelle, Hong Qin, Hermine Agis, Renate Kain, Christian Hengstenberg, Roza Badr Eslam, Diana Bonderman

**Affiliations:** 1 Department of Internal Medicine II, Department of Cardiology, Medical University of Vienna, Waehringer Guertel 18-20, 1090 Vienna, Austria; 2 Department of Internal Medicine I, Department of Oncology, Medical University of Vienna, Vienna, Austria; 3 Clinical Institute of Pathology, Medical University of Vienna, Vienna, Austria

**Keywords:** Cardiac amyloidosis, Echocardiography, Effusion, Right ventricle, Speckle tracking imaging, Strain, Outcome

## Abstract

**Background:**

Pericardial and pleural effusion are common findings in patients with cardiac amyloidosis (CA). It is not known, whether effusions correlate with right ventricular (RV) function in these patients. Furthermore, data on the prognostic significance of pleural and pericardial effusion in CA is scarce.

**Methods:**

Patients with transthyretin (ATTR) and light chain (AL) CA were included in a clinical registry. All patients underwent transthoracic echocardiography at baseline. The presence of pericardial and pleural effusion was determined in every patient. The clinical endpoint was defined as cardiac death or heart failure hospitalization.

**Results:**

In total, 143 patients were analysed. Of these, 85 patients were diagnosed with ATTR and 58 patients with AL. Twenty-four patients presented with isolated pericardial effusion and 35 with isolated pleural effusion. In 19 patients, both pericardial and pleural effusion were found and in 65 patients no effusion was present at baseline. The presence of pleural effusion correlated well with poor RV function, measured by global RV free-wall strain (*p* = 0.007) in patients with AL, but not in ATTR. No such correlation could be found for pericardial effusion in either amyloidosis subtype. Patients with AL presenting with pleural effusion had worse outcomes compared to patients with pericardial effusion alone or no effusion at baseline. In the ATTR group, there was no difference in outcomes according to presence and type of effusion.

**Conclusion:**

More than 50% of patients with CA presented with pleural and/or pericardial effusions. While pleural effusion was clearly associated with poor RV function in AL, we were not able to detect this association with pericardial effusion.

**Electronic supplementary material:**

The online version of this article (10.1007/s00392-020-01698-7) contains supplementary material, which is available to authorized users.

## Introduction

Pleural and pericardial effusions are often seen in patients with heart failure (HF) and are generally attributed to right heart failure [1]. Effusions are also especially common and occur in more than 50% of patients with cardiac amyloidosis (CA) [2, 3]. However, data on the exact prevalence and relevance of effusions in CA are scarce and descriptions are largely limited to small single centre studies and case reports.[4, 5].

### Pericardial effusions in cardiac amyloidosis

Ultrasound is an ideal imaging modality to detect pericardial and pleural effusion as it is more sensitive than physical examination or chest X-ray [6]. It is, therefore, suggested in the current guidelines as a standard assessment in the evaluation of patients with HF and should, therefore, also be part of a detailed echocardiographic examination in patients with CA [7]. One study reported on the predictive effect of pericardial effusion in patients with light chain amyloidosis (AL); however, the studied cohort was small (*n* = 31) and only analysed the effects of effusion on all-cause death rather than cardiac death [8, 9]. There has recently been evidence that pericardial effusion may also be a sign of myocardial edema following local amyloid infiltration and consecutive inflammation in patients with AL and ATTR [10, 11]. This may be especially relevant in AL due to cytotoxicity of light chain amyloid fibrils [12]. On this note, Riduoani et al. were recently able to show that cardiac magnetic resonance imaging (CMR) could detect local inflammation using T2 weighted sequences and may be useful in distinguishing between ATTR and AL [13]. It has also previously been proposed that in patients with AL, pleural effusion may not only result from right HF, but also from primary pleural infiltration causing consecutive fluid secretion [14]. Another potential mechanism could be a fluid shift resulting from a decrease in intravascular oncotic pressure due to an increased renal loss or reduced production of serum proteins. The exact mechanism of the development of pericardial and pleural effusions in CA even in patients with presumably preserved left and right ventricular (LV, RV) function, however, remains unclear.

We conducted this study to further shed light on pericardial and pleural effusions in AL and ATTR with cardiac involvement and their impact on cardiac outcome.

## Methods

### Study population

We consecutively included patients with AL and ATTR amyloidosis in our prospective clinical registry at the Medical University of Vienna. Diagnosis as well as follow-up were performed and documented at our dedicated CA outpatient clinic. Visits were routinely scheduled every 6 months or more frequently when appropriate, as judged by the clinician. When necessary, patients were admitted to the cardiology ward for intensified treatment. All patients gave written informed consent before study inclusion. The study protocol complies with the Declaration of Helsinki and was approved by the local Ethics Committee of the Medical University of Vienna, Austria (Ethics committee identification number: 796/2010).

### Diagnosis of cardiac amyloidosis

In the years preceding 2016, the diagnosis of ATTR was made by endomyocardial biopsy (EMB). At least five tissue samples were taken from the LV myocardium (Bipal® biopsy foceps Cordis® Corporation, Bridgewater, NJ). Specimens were then fixed in formaldehyde, embedded in paraffin and subsequently stained with congo-red dye to detect interstitial amyloid deposits. Additionally, tissue samples were examined under polarized light to reveal green birefringence. When amyloid fibrils were found, inmmunohistochemical analysis was performed to further characterize amyloid type according to local standard established procedures (AmY-kit amyloid antibodies, Martinsried, Germany).

Following the landmark publication of Gillmore et al. in 2016, which presented a non-invasive diagnostic algorithm for the diagnosis of ATTR using serum and urine light chain analysis as well as bone scintigraphy, transthoracic echocardiography (TTE) and/or CMR, EMB was only performed when non-invasive test results, where ambiguous or unclear [15]. CMR was performed including gadolinium contrast application, T1 mapping and calculation of extracellular volume, as previously described [16, 17].

The diagnosis of AL was either made by EMB or by extramyocardial biopsy. To confirm the presence of cardiac involvement, at least one of the following features had to be present: (1) LV hypertrophy with an interventricular septum (IVS) thickness of > 12 mm and/or presence of apical sparing determined by TTE or (2) elevated cardiac biomarkers [18, 19].

### Definitions of clinical endpoints

Clinical outcomes were documented by follow-up at our outpatient clinic, as well as by phone calls and by screening medical records in our electronic hospital record system. The clinical endpoints were defined as cardiac death and death from any cause. Further endpoints were death from any cause and a combined endpoint of cardiac death or HF hospitalization. In the case of a clinical event, local and external records were carefully screened and cause of death was reviewed by a clinical adjudication committee of board certified cardiology specialists (D.B., R.B.). HF hospitalization was defined as an event leading to sudden dyspnea, weight gain, peripheral edema and requiring admission to hospital and/or intravenous diuretic therapy.

### Baseline assessment and transthoracic echocardiography

Baseline assessment was performed at our CA outpatient clinic and included demographic, clinical, laboratory and imaging parameters.

All TTE exams included standard imaging parameters, as well as an extended protocol performed by experienced and certified specialists on high-end machines (GE Vivid 95 and Vivid 7; GE Healthcare, Wauwatosa, WI, USA). Images were acquired and measurements were taken according to current guideline recommendations [20–22].

Presence and size of pericardial effusion was assessed in a subcostal four-chamber view. When imaging quality was suboptimal from a subcostal approach, an apical four-chamber view was used to determine the presence of pericardial effusion. Assessment of pleural effusion was performed bilaterally in every patient. Additionally, the presence of effusion was confirmed by chest X-ray or CMR in patients who underwent CMR for the diagnosis of CA.

Speckle tracking imaging was performed after image acquisition on a modern offline clinical workstation equipped with dedicated software (EchoPAC; GE Healthcare, Wauwatosa, WI, USA). The global longitudinal strain of the LV (LV-GLS) was measured by tracking the myocardium in an optimized apical three- four- and two-chamber view. The global longitudinal strain of the RV (RV-GLS) was measured in the free lateral RV wall in an optimized apical four-chamber view. All obtained TTE parameters are shown in Table 1.

**Table 1:** Baseline characteristics for patients with amyloidosis with and without pericardial and/or pleural effusion

	Total population (*n* = 143)	No effusion (*n* = 65)	Pericardial and/or pleural effusion (*n* = 78)	*p*-value
*Clinical parameters*				
Age, years	73.0 (66.0–78.0)	78.0 (67.0–78.0)	74.0 (63.0–80.0)	0.697
Male sex	107 (75)	51 (79)	56 (72)	0.360
BMI, kg/m^2^	25.6 (23.2–28.4)	26.0 (23.0–29.0)	26.0 (23.0–28.0)	0.680
Systolic BP, mmHg	126.5 (113.0–140.0)	125.0 (113.0–140.0)	128.0 (113.0–139.0)	0.654
Diastolic BP, mmHg	75.0 (70.0–85.0)	77.0 /70.0–87.0)	74.0 (69.0–84.0)	0.354
Heart rate, bpm	75.0 (66.5–87.0)	76.0 (68.0–87.0)	74.0 (65.0–87.0)	0.566
*NYHA class*				**0.018**
NYHA I	14 (9.8)	10 (15.4)	4 (5.1)	
NYHA II	61 (42.7)	33 (50.8)	28 (35.9)	
NYHA III	64 (44.8)	21 (32.3)	43 (55.2)	
NYHA IV	4 (2.7)	1 (1.5)	3 (3.8)	
*Comorbidities*				
Polyneuropathy	56 (39.2)	30 (46.2)	26 (33.3)	0.118
Diabetes mellitus	20 (14.0)	12 (18.5)	8 (10.3)	0.168
Atrial fibrillation	65 (45.5)	27 (41.5)	38 (48.7)	0.352
Coronary artery disease	29 (20.3)	11 (16.9)	18 (23.1)	0.362
Intracardiac device	19 (23.3)	8 (12.3)	11 (14.1)	0.753
*Concomitant medication*				
AT II antagonist	35 (24.5)	19 (29.2)	16 (20.5)	0.227
ACE inhibitor	37 (25.9)	13 (20.0)	24 (30.8)	0.143
Beta-blocker	76 (53.1)	32 (49.2)	44 (56.4)	0.392
Aldosterone antagonist	64 (44.8)	20 (30.8)	44 (56.4)	**0.002**
Loop diuretics	91(63.6)	30 (46.2)	61 (78.2)	** < 0.001**
Oral anticoagulant	67 (46.9)	29 (44.6)	38 (48.7)	0.624
*Echocardiography parameters*				
LA length, mm	61.0 (55.0–68.0)	60.0 (55.0–66.0)	63.0 (55.0–68.0)	0.147
RA length, mm	59.0 (53.0–64.3)	58.0 (53.0–63.0)	60.0 (52.0–66.0)	0.236
LVEDD, mm	41.0 (37.0–46.0)	42.0 (38.0–47.0)	41.0 (34.0–46.0)	0.060
LVEF, %	45.0 (46.0–64.3)	52.0 (47.0–65.0)	55.0 (46.0–64.0)	0.993
LV-GLS, -%	12.0 (15.0–9.0)	13.7 (16.8–10.0)	11.3 (13.8–8.6)	**0.007**
IVS, mm	19.0 (16.0–22.0)	18.0 (16.0–22.0)	20.0 (17.0–23.0)	0.155
RVEDD, mm	33.0 (28.0–38.0)	34.0 (29.0–38.0)	33.0 (28.0–38.0)	0.496
RV-TDI, m/s	0.11 (0.09–0.14)	0.12 (0.09–0.14)	0.11 (0.09–0.13)	0.485
TAPSE, mm	16.0 (13.0–19.0)	17.0 (14.0–20.0)	15.0 (12.0–18.0)	0.055
RV-GLS, -%	16.3 (20.7–11.3)	17.7 (23.0–13.3)	14.0 (18.5–10.0)	**0.004**
TR velocity, m/s	2.9 (2.6–3.3)	2.9 (2.5–3.2)	2.9 (2.7–3.3)	0.446
sPAP, mmHg	43.0 (37.0–58.0)	43.0 (32.0–55.0)	43.0 (38.0–58.0)	0.528
IVC diameter, mm	20.0 (17.0–23.0)	19.0 (14.0–22.0)	20.0 (18.0–24.0)	0.237
*Laboratory parameters*				
NT-pro BNP, pg/mL	2815.0 (1412.0–7173.0)	1635.0 (827.0–3549.0)	4268.5 (2048.0–8866.0)	**< 0.001**
Troponin T, ng/L	52.0 (31.0–96.5)	38.0 (22.0–56.0)	68.0 (43.5–118.0)	**< 0.001**
Hemoglobin, g/dL	12.7 (11.3–14.2)	12.9 (11.3–14.4)	12.7 (11.1–14.1)	0.377
Creatinine, mg/dL	1.2 (1.0–1.6)	1.3 (0.9–1.6)	1.2 (1.0–1.6)	0.813
eGFR, mL/min	51.7 (38.3–63.1)	52.3 (40.7–72.4)	51.0 (37.9–59.1)	0.327
ASAT, U/L	28.0 (22.0–34.0)	27.0 (22.0–33.0)	29.0 (23.0–35.0)	0.102
ALAT, U/L	24.0 (17.0–33.0)	23.0 (17.0–29.0)	24.0 (17.0–35.0)	0.424
Albumin, g/L	40.5 (35.0–44.1)	43.3 (38.3–45.2)	38.7 (33.0–42.4)	**0.002**
GGT, U/L	65.0 (32.0–137.0)	42.0 (26.0–126.0)	79.0 (46.0–140.0)	**0.033**
LDH, U/L	231.0 (197.0–282.0)	224.0 (190.0–249.0)	241.0 (206.0–292.5)	**0.017**
CRP, mg/dL	0.3 (0.1–1.1)	0.2 (0.1–0.9)	0.4 (0.1–1.1)	0.060
*Cardiac magnetic resonance imaging parameters*				
IVS, mm	19.0 (15.5–22.0)	18.0 (15.0–22.0)	17.0 (19.0–22.0)	0.197
LVEF, %	57.0 (49.0–63.0)	57.0 (45.0–65.0)	57.0 (50.0–62.0)	0.714
LV-ECV, %	46.0 (40.0–55.5)	45.0 (36.0–52.0)	49.0 (41.0–58.0)	0.090
RVEF, %	49.5 (40.1–60.5)	49.0 (42.0–61.0)	51.0 (38.0–59.0)	0.556

Continuous variables are given in median and interquartile range, categorical data are shown as numbers and percentages

*BMI* body mass index, *BP* blood pressure, *bpm* beats per minute, *NYHA* New York Heart Association, *CAD* coronary artery disease, *PM* pace maker, *ICD* intracardiac defibrillator, *AT II* angiotensin II, *ACE* angiotensin converting enzyme, *LA* left atrium, *RA* right atrium, *LVEDD* left ventricular end-diastolic diameter, *LVEF* left ventricular ejection fraction, *IVS* intraventricular septum, *RVEDD* right ventricular end-diastolic diameter, *RV-TDI* right ventricular tissue Doppler index, *TAPSE* tricuspid annular plane systolic excursion, *RV-GLS* right ventricular global longitudinal strain, *TR* tricuspid regurgitation *sPAP* systolic pulmonary artery pressure, *IVC* inferior vena cava, *NT-pro BNP* N-terminal pro brain natriuretic peptide, *eGFR* estimated glomerular filtration rate, *ASAT* aspartate aminotransferase, *ALAT* alanine aminotransferase, *GGT* gamma glutamyltransferase, *LDH* lactate dehydrogenase, *CRP* C-reactive protein, *LV-ECV* left ventricular extra cellular volume, *RVEF* right ventricular ejection fraction

Pericardial effusion size was measured at the location of maximum fluid accumulation. Pericardial effusions were defined as hemodynamically significant according to current guidelines [4], i.e., presence of swinging heart, early diastolic collapse of the RV, abnormal ventricular septal motion, exaggerated respiratory variability (> 25%) in mitral inflow velocity, or when the patient was clinically compromised (cardiogenic shock).

### Statistical analysis

Continuous data are shown as median values and inter-quartile range (IQR). Categorical variables are expressed as numbers and percentages. Analysis of variance (ANOVA), Student’s *T*-test and Chi square test were used as appropriate to determine statistically significant differences in baseline variables. Univariable Cox regression analysis was applied to test baseline variables for effects on the primary endpoint of cardiac death or HF hospitalization. Variables which significantly predicted outcome were then entered in a stepwise forward multivariable Cox regression model within respective categories to adjust for potential confounding effects. Kaplan–Meier plots with respective log-rank tests were computed to assess the time-dependent discriminative power of effusion type on endpoints. A *p*-value < 0.05 was considered as statistically significant. All analyses were performed using SPSS 24 (IBM Corp. NY, USA).

## Results

### Study cohort

In total, 169 patients with cardiac AL or ATTR amyloidosis were included in our clinical amyloidosis registry between March 2012 and January 2019. However, only 143 patients had sufficient echocardiographic image quality of both ventricles to be included in this study. Of these, 85 patients (59.4%) presented with ATTR and 58 (40.6%) with AL. Diagnosis was confirmed by EMB in 19 patients with AL (13.3%) and 31 patients with ATTR (21.7%). In 37 patients, the diagnosis of CA was made using a combination of non-cardiac biopsy and imaging (AL = 33, ATTR = 4). All other were diagnosed by imaging alone. CMR was performed in 113 patients (AL = 47, ATTR = 66).

### Baseline characteristics and clinical presentation

Detailed baseline characteristics of the study population according to presence of pericardial and/or pleural effusion are shown in Table 1.

Patients with effusions (*n* = 78) presented with more severe clinical symptoms as described by New York Heart Association (NYHA) class (*p* = 0.018) and were more commonly already on loop diuretics (*p* < 0.001) and/or aldosterone antagonists (*p* = 0.002) at baseline. Left ventricular global longitudinal strain (LV-GLS) as well as RV free wall strain (RV-GLS) were more severely impaired in patients with pericardial or pleural effusions with *p*-values of 0.007 and 0.004, respectively.

Notably, patients with effusions not only showed higher levels of cardiac biomarkers (median NT-pro BNP 4268.5 pg/ml (IQR 2048.0–8866.0 pg/mL) versus 1635.0 pg/ml (IQR 827.0–3549.0 pg/mL), *p* < 0.001 and median troponin T 68.0 ng/L (IQR43.5–118.0 ng/L) versus 28.0 ng/L (IQR 22.0–56.0 ng/L), *p* < 0.001), but also higher levels of gamma glutamyltransferase (GGT) of 79.0 U/L (IQR 46.0–140.0 U/L) versus 42.0 U/L [(IQR26.0–126.0 U/L), *p* = 0.033] and lower levels of serum albumin of 38.7 g/L (IQR 33.0–42.4 g/L) versus 43.0 g/L [(IQR 38.3–45.2 g/L), *p* = 0.002]. Furthermore, median serum lactate dehydrogenase was higher in patients presenting with pericardial and/or pleural effusions [(241.0 U/L (IQR 206.0–292.5 U/L) versus 224.0 U/L (IQR 190.0–249.0 U/L), *p* = 0.017].

### Pericardial and pleural effusion

At baseline, 35 patients (24.5%) presented with isolated pleural effusion, 24 patients (16.8%) with isolated pericardial effusion and 19 patients (13.3%) with both pleural and pericardial effusion. Pleural effusions detected by echocardiography could only be confirmed by chest X-ray in 76.2% of all patients. Table 2 shows a detailed description of types of effusions and their distribution in patients with AL versus ATTR. In general, pericardial effusions were small with a medium diameter of 7.1 mm (IQR 5.2–9.7 mm) and none were hemodynamically compromising.

**Table 2:** Effusions in patients with cardiac light-chain (AL) versus transthyretin amyloidosis (ATTR)

	Total population (*n* = 143)	AL (*n* = 58)	ATTR (*n* = 85)
No effusion	65 (45.5)	21 (36.2)	44 (51.8)
Isolated pleural effusion	35 (24.5)	16 (27.6)	19 (22.4)
Isolated pericardial effusion,	24 (16.8)	12 (20.7)	12 (14.1)
Pleural and pericardial effusion	19 (13.3)	9 (15.5)	10 (11.8)

Values are given as numbers and percentages.

### Effusions in light chain amyloidosis

**Fig. 1:**
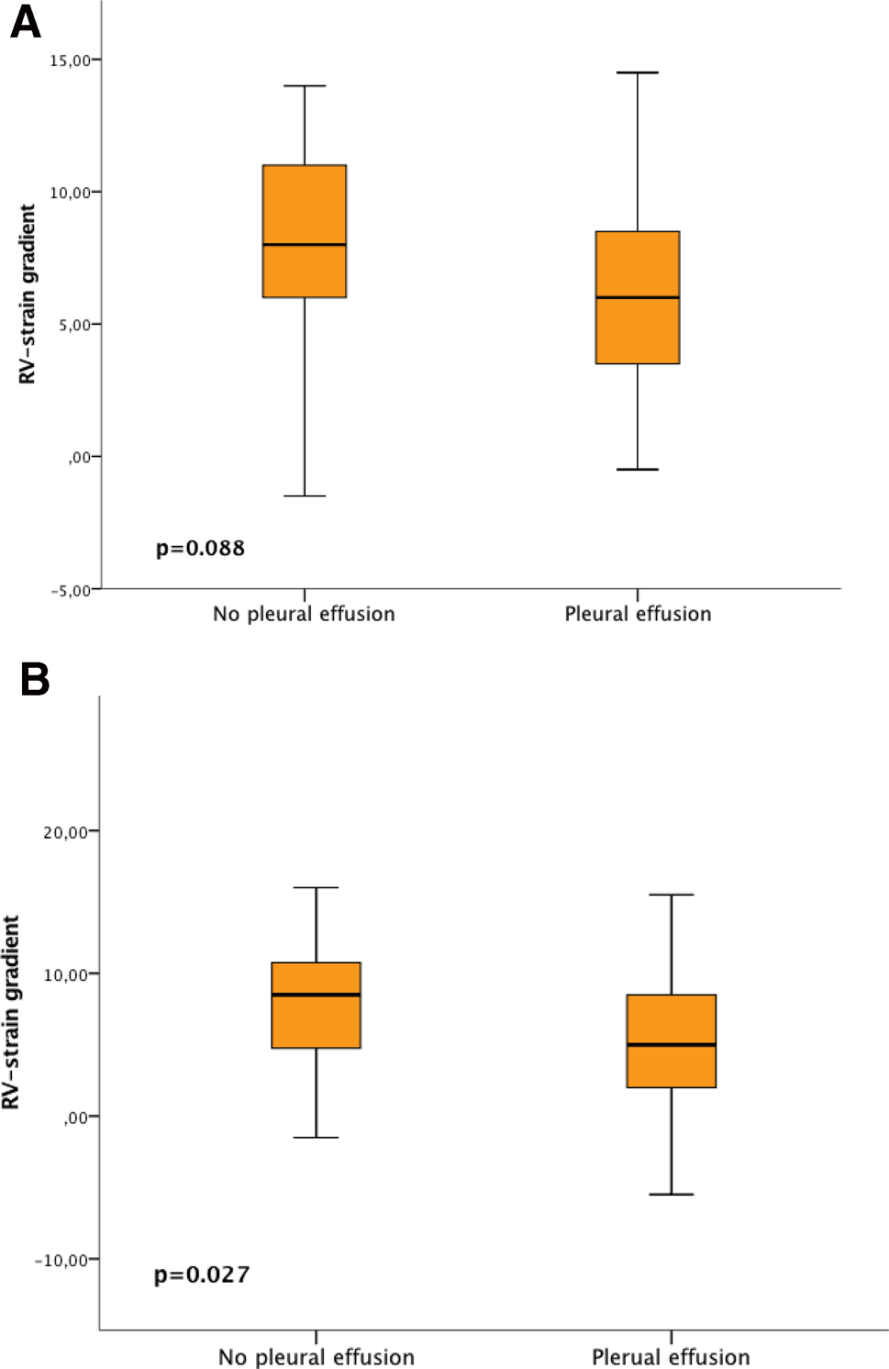
2D strain in the basal and mid segments compared to the apical segments of the right ventricle in light-chain amyloidosis (**a**) and ATTR (**b**) in patients with and without pleural effusions

When analysing parameters associated with right heart dimensions and function, we found that patients with AL presenting with pleural effusions at baseline had more impaired RV function, which was reflected by RV-GLS, TAPSE and RV-TDI. There was no difference in RV strain when comparing the basal- and mid segments with the apical segments of the RV (Fig. 1a). Patients with pleural effusions had higher amyloid burden, seen as increased interventricular wall thickness (IVS) and LV extra cellular volume (LV-ECV) resulting in impaired contractility as measured by LV-GLS. Interestingly, not only cardiac biomarkers such as serum NT-pro BNP and troponin T, but also GGT and CRP levels were higher in patients with pleural effusions (Table 3).

**Table 3:** Baseline echocardiographic and laboratory parameters of patients with light chain amyloidosis (*n* = 58) with and without pleural effusion* (extracted)

	No pleural effusion (*n* = 33)	Pleural effusion (*n* = 25)	*p*-value
*Echocardiography parameters*			
LA length mm	60.0 (56.0–67.0)	60.0 (52.0–64.0)	0.665
LVEDD, mm	46.0 (43.0–48.0)	35.0 (32.0–46.0)	** < 0.001**
LVEF, %	61.0 (51.0–70.0)	56.0 (46.0–65.0)	0.626
LV-GLS, -%	14.8 (19.10–14.0)	11.3 (14.8–8.3)	**0.003**
IVS, mm	16.0 (14.0–18.0)	19.0 (16.0–22.0)	0.170
RV diameter, mm	34.0 (30.0–37.0)	30.0 (26.0–36.0)	**0.044**
RA length, mm	59.0 (51.0–64.0)	58.0 (52.0–61.0)	0.826
RV strain basal, -%	16.5 (20.0–14.0)	13.0 (18.0–11.0)	**0.009**
RV strain mid, -%	18.0 (21.0–15.0)	14.0 (17.0–11.0)	**0.007**
RV strain apical, -%	19.0 (23.0–15.0)	14.0 (18.0–12.0)	**0.013**
RV- GLS, -%	18.0 (21.3–15.0)	14.0 (17.7–11.0)	**0.007**
RV- TDI, m/s	0.14 (0.13–0.16)	0.11 (0.09–0.12)	0.009
TAPSE, mm	18.0 (15.0–22.0)	14.0 (13.0–16.0)	**0.001**
TR velocity, m/s	3.0 (2.6–3.7)	2.8 (2.6–3.1)	0.251
sPAP, mmHg	51.0 (35.0–67.0)	42.5 (39.0–50.0)	0.354
IVC diameter, mm	19.0 (14.0–25.0)	19.0 (18.0–23.0)	0.594
*Laboratory parameters*			
NT-pro BNP, pg/mL	2292.0 (1121.0–4118.0)	8866.0 (4751.0–17,103.0)	**< 0.001**
Troponin T, ng/L	41.0 (21.0–73.5)	112.0 (65.0–232.0)	**0.001**
Gamma GT, U/L	36.0 (21.0–77.0)	117.0 (56.0–194.0)	**< 0.001**
ASAT, U/L	23.0 (18.0–28.0)	27.0 (20.0–43.0)	0.094
ALAT, U/L	21.0 (17.0–26.0)	22.0 (15.0–43.0)	0.994
Albumin, U/L	39.2 (30.6–42.6)	33.9 (28.5–40.3)	0.253
CRP, mg/dl	0.2 (0.1–1.0)	0.9 (0.3–1.9)	**0.021**
eGFR, mL/min/1.73m^2^	47.8 (33.3–61.5)	40.5 (22.7–55.9)	0.316
*Cardiac magnetic resonance imaging parameters*			
IVS, mm	14.0 (12.0–26.0)	19.0 (15.0–21.0)	**0.005**
LVEF, %	63.0 (53.0–66.0)	61.0 (57.0–65.0)	0.572
LV-ECV, %	40.0 (32.0–43.0)	48.0 (41.0–59.0)	**0.003**
RVEF, %	57.0 (48.0–62.0)	56.0 (45.0–5620)	0.405

Continuous variables are given in mean and interquartile range

*LA* indicates left atrium, *LVEDD* left ventricular end-diastolic diameter, *LVEF* left ventricular ejection fraction, *IVS* interventricular septum, *RV* right ventricle, *RA* right atrium, *RV-GLS* right ventricular global longitudinal strain, *TDI* tissue Doppler index, *TAPSE* tricuspid annular plane systolic excursion, *TR* tricuspid regurgitation, *sPAP* systolic pulmonary artery pressure, *IVC* inferior vena cava, *NT-pro BNP* N-terminal pro brain natriuretic peptide, *Gamma GT* gamma glutamyltransferase, *ASAT* aspartate aminotransferase, *ALAT* alanine aminotransferase, *CRP* C-reactive protein, *eGFR* estimated glomerular filtration rate calculated by the modification of diet in renal disease (MDRD) formula, *LV-ECV* left ventricular extra cellular volume, *RVEF* right ventricular ejection fraction

By contrast, AL patients presenting with pericardial effusions only showed significantly more impaired strain in the basal and mid regions of the RV. Apical RV regions also showed more impaired function, even though this difference was not statistically significant (supplementary table S1). Notably, there was no difference in cardiac biomarkers between the two groups.

### Effusions in transthyretin amyloidosis

In patients diagnosed with ATTR, the presence of pleural effusions was not accompanied by more impaired RV strain values or other parameters of RV function. However, when comparing the basal and mid-segments of the RV to the apical segments, we were able to detect that the more apical segments showed better RV function (RV-apical sparing, Fig. 1b). Similarly, there was no statistically significant association between the presence of pleural effusion and LV-function or degree of LV amyloid infiltration seen in CMR. Nevertheless, serum levels of cardiac biomarkers were significantly higher in patients with pleural effusions compared to those without. Notably, albumin levels were lower in patients presenting with pleural effusions (Table 4). Supplementary table S2 shows, that there is no association between RV function and pericardial effusion in patients with ATTR.

**Table 4:** Baseline echocardiographic and laboratory parameters of patients with transthyretin amyloidosis (*n* = 85) with and without pleural effusion*

	No pleural effusion (*n* = 56)	Pleural effusion (*n* = 29)	*p*-value
*Echocardiography parameters*			
LA length, mm	60.0 (54.0–63.0)	66.0 (60.0–71.0)	**0.036**
LVEDD, mm	40.0 (38.0–44.0)	42.0 (38.0–45.0)	0.110
LVEF, %	52.0 (46.0–62.0)	54.0 (45.0–63.0)	0.516
LV-GLS, -%	12.1 (15.3–8.9)	11.2 (13.1–8.6)	0.052
IVS, mm	20.0 (17.0–23.0)	20.0 (17.0–24.0)	0.790
RV diameter, mm	32.0 (28.0–37.0)	36.0 (31.0–42.0)	0.401
RA length, mm	58.0 (53.0–63.0)	63.0 (59.0–68.0)	**0.017**
RV strain basal, -%	17.0 (21.0–11.5)	12.5 (19.5–10.0)	0.242
RV strain mid, -%	17.0 (21.0–12.0)	14.0 (19.0–10.0)	0.308
RV strain apical, -%	17.0 (21.0–12.0)	16.5 (20.5–10.5)	0.795
RV- GLS, -%	17.0 (20.8–12.0)	13.8 (19.5–10.2)	0.399
RV- TDI, m/s	0.10 (0.08–0.13)	0.10 (0.09–0.12)	0.965
TAPSE, mm	16.0 (12.0–19.0)	16.0 (11.0–20.0)	0.892
TR velocity, m/s	2.9 (2.6–3.2)	2.9 (2.6–3.3)	0.783
sPAP, mmHg	42.0 (35.0–55.0)	50.0 (37.0–61.0)	0.343
IVC diameter, mm	18.0 (15.0–22.0)	22.0 (20.0–25.0)	**0.010**
*Laboratory parameters*			
NT-pro BNP, pg/mL	1574.5 (846.4–3367.0)	3908.0 (2048.0–7173.0)	**< 0.001**
Troponin T, ng/L	39.0 (29.0–64.0)	68.0 (40.0–79.0)	**0.049**
Gamma GT, U/L	66.0 (28.5–140.0)	72.0 (45.0–140.0)	0.384
ASAT, U/L	30.0 (24.0–34.0)	31.0 (25.0–35.0)	0.366
ALAT, U/L	25.5 (20.0–33.0)	23.0 (17.0–35.0)	0.450
Albumin, U/L	43.6 (40.4–46.3)	38.7 (35.9–42.9)	**0.003**
CRP, mg/dl	0.2 (0.1–0.7)	0.3 (0.2–1.2)	0.096
eGFR, mL/min/1.73m^2^	56.0 (45.7–74.7)	47.5 (40.6–56.9)	0.092
*Cardiac magnetic resonance imaging parameters*			
IVS, mm	18.0 (15.0–22.0)	20.0 (18.0–23.0)	0.667
LVEF, %	55.0 (42.0–60.0)	51.0 (46.0–57.0)	0.098
LV-ECV, %	49.0 (43.0–56.0)	51.0 (42.0–57.0)	0.173
RVEF, %	47.0 (39.0–60.0)	48.0 (37.0–55.0)	0.474

Continuous variables are given in mean and interquartile range

*LA* indicates left atrium, *LVEDD* left ventricular end-diastolic diameter, *LVEF* left ventricular ejection fraction, *IVS* interventricular septum, *RV* right ventricle, *RA* right atrium, *RV-GLS* right ventricular global longitudinal strain, *TDI* tissue Doppler index, *TAPSE* tricuspid annular plane systolic excursion, *TR* tricuspid regurgitation, *sPAP* systolic pulmonary artery pressure, *IVC* inferior vena cava, *NT-pro BNP* N-terminal pro brain natriuretic peptide, *Gamma GT* gamma glutamyltransferase, *ASAT* aspartate aminotransferase, *ALAT* alanine aminotransferase, *CRP* C-reactive protein, *eGFR* estimated glomerular filtration rate calculated by the modification of diet in renal disease (MDRD) formula, *LV-ECV* left ventricular extra cellular volume, *RVEF* right ventricular ejection fraction

*10 patients of the shown population had additional pericardial effusion.

### Left and right heart function in patients with AL versus ATTR

LV hypertrophy was more pronounced in patients with ATTR than those who had been diagnosed with AL with a mean IVS thickness of 20 mm versus 17 mm, respectively. LV function was better in patients with AL as indicated by LV-GLS (*p* = 0.010), as well as LV ejection fraction (LVEF) measured by CMR (*p* < 0.001). Interestingly, RV function measured using the volumetric method of RV ejection fraction by CMR was also better in patients with AL (*p* = 0.007), while RV function parameters reflecting RV contractility, such as RV-GLS and TAPSE did not show any differences between patients with AL and ATTR (*p* = 0.562 and 0.298, respectively).

### Outcomes according to presence and localization of effusion

After a median follow-up time of 19.0 months (IQR 9.5–32.5), 42 patients had died (29.4%). In more detail, 28 patients with AL (48.3% of the total AL population) and 14 patients with ATTR (16.5% of the total ATTR population) died. During follow-up, 26 (61.9%) could be classified as cardiac deaths. In seven patients, the exact cause of death could not be certified, due to lack of medical records or information from relatives and were, therefore, classified as all-cause death.

**Fig. 2:**
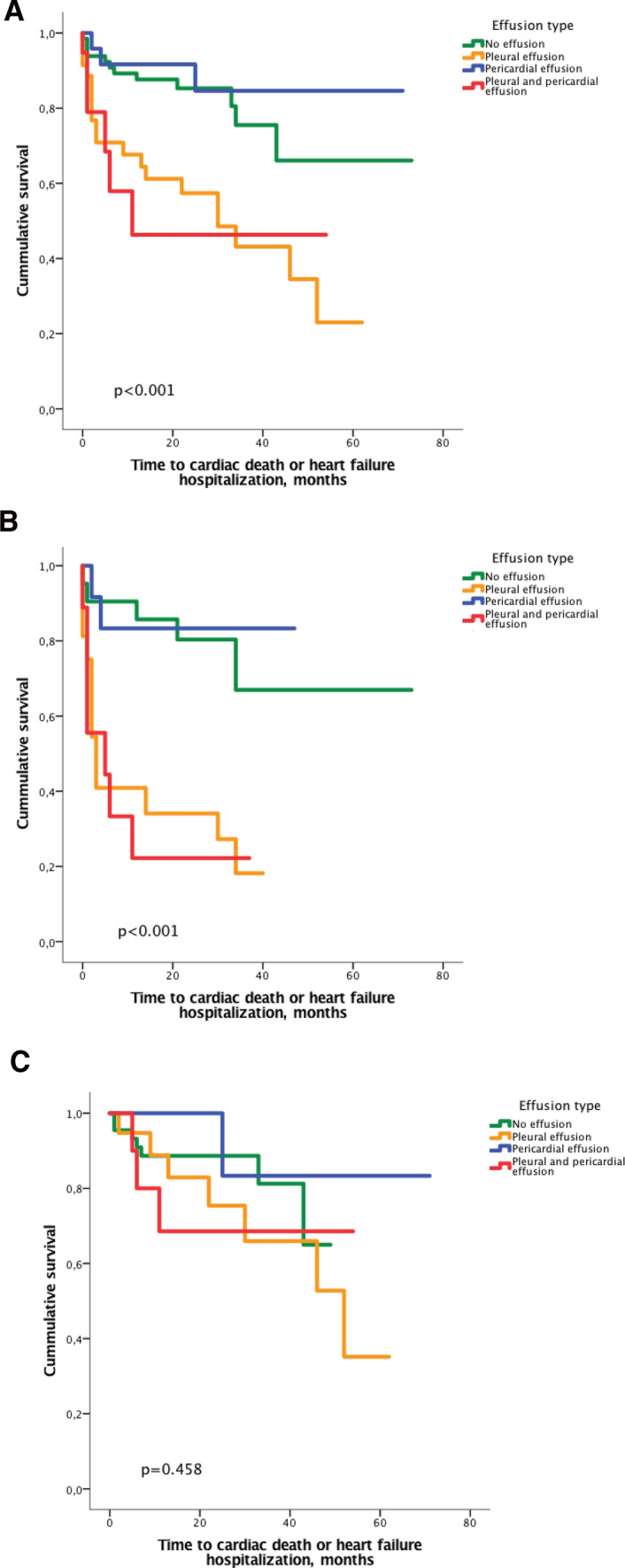
Kaplan–Meier plot showing time to cardiac death or heart failure in all patients with cardiac amyloidosis (*n* = 143, **a**), patients with light chain amyloidosis (*n* = 58, **b**) and transthyretin amyloidosis (*n* = 85, **c**) according to the presence of pericardial and/or pleural effusion at baseline

Kaplan–Meier analysis showed that among the total population of cardiac amyloidosis, patients with pleural effusions had worse outcomes than patients with no effusions or isolated pericardial effusions. This was irrespective of concomitant presence of pericardial effusion in addition to pleural effusion. Furthermore, the presence of isolated pericardial effusion did not have an impact on outcome. When separating the studied cohort by amyloid subtype, we saw that effusion type was predictive for outcome in patients with AL, but not in ATTR (Fig. 2). In addition, we used univariable Cox regression models to calculate hazard ratios (HR) for cardiac death for all baseline parameters in patients with AL. We found that the presence of pleural effusion predicted adverse outcome with a HR of 5.604 [(2.334–13.456), *p* < 0.001] alongside with NYHA class, NT-pro BNP and troponin T. Among echocardiography parameters, LV global longitudinal strain (LV-GLS), LV end diastolic diameter (indexed to body surface area), interventricular septum thickness, and RV tissue Doppler index (RV-TDI) predicted outcome in the univariable model. After adjusting for confounding factors within respective categories of baseline characteristics, only NYHA class, LV-GLS and NT-pro BNP remained predictive in the multivariable model (Table 5).

**Table 5:** Univariable and multivariable Cox regression analysis of baseline characteristics in patients with light chain amyloidosis calculated for the endpoint of cardiac death or heart failure hospitalization

	Univariable HR (95%CI)	*p*-value	Multivariable HR (95%CI)	*p*-value
*Clinical variables*				
Age, years	0.999 (0.997–1.001)	0.270		
Male sex	0.465 (0.214–1.013)	0.054		
BMI, kg/cm^2^	0.986 (0.933–1.043)	0.628		
Systolic BP, mmHg	0.995 (0.973–1.017)	0.630		
Diastolic BP, mmHg	0.997 (0.972–1.022)	0.815		
Heart rate, bpm	1.005 (0.983–1.029)	0.651		
NYHA class	4.367 (2.186–8.723)	**< 0.001**	2.628 (1.613–4.282)	**< 0.001**
Pleural effusion	5.604 (2.334–13.456)	**< 0.001**	0.810 (0.356–1.845)	0.616
Pericardial effusion	0.970 (0.432–2.180)	0.942		
*Comorbidities*				
Diabetes mellitus	1.235 (0.462–3.301)	0.674		
Atrial fibrillation	0.685 (0.294–1.596)	0.380		
Coronary artery disease	1.619 (0.551–4.756)	0.381		
Intracardiac device	1.544 (0.530–4.494)	0.426		
*Echocardiography parameters*				
LA length*, mm	0.990 (0.951–1.030)	0.609		
RA length*, mm	1.004 (0.963–1.047)	0.854		
LVEDD*, mm	0.887 (0.838–0.939)	** < 0.001**	0.988 (0.911–1.071)	0.767
LVEF, %	1.010 (0.976–1.046)	0.556		
LV-GLS, -%	1.113 (1.020–1.215)	**0.017**	1.133 (1.018–1.261)	**0.022**
IVS, mm	1.115 (1.024–1.214)	**0.013**	1.071 (0.943–1.215)	0.291
RVEDD*, mm	0.965 (0.880–1.057)	0.441		
RV-TDI, m/s	0.528 (0.301–0.927)	**0.026**	0.659 (0.328–1.326)	0.243
TAPSE, mm	0.947 (0.864–1.039)	0.248		
RV-GLS, -%	1.080 (0.988–1.182)	0.091		
TR velocity, m/s	1.232 (0.608–2.500)	0.563		
sPAP, mmHg	1.011 (0.983–1.040)	0.428		
IVC, mm	0.958 (0.877–1.046)	0.335		
*Laboratory parameters*				
NT-pro BNP**pg/mL	4.959 (2.023–12.158)	**0.005**	7.212 (2.248–23.136)	**0.001**
Troponin T**ng/L	4.282 (1.667–11.003)	**0.003**	2.916 (0.779–10.906)	0.112
Hemoglobin, mg/dL	0.996 (0.833–1.192)	0.968		
Creatinine, mg/dL	0.866 (0.690–1.086)	0.213		
eGFR, mL/min	0.992 (0.978–1.005)	0.241		
ASAT, U/L	1.008 (0.997–1.020)	0.154		
ALAT, U/L	1.010 (0.997–1.022)	0.139		
Albumin, g/L	0.968 (0.930–1.008)	0.113		
GGT, U/L	1.001 (1.000–1.002)	0.172		
LDH, U/L	1.003 (1.000–1.006)	0.080		
CRP, mg/dL	1.033 (0.898–1.187)	0.653		

*BMI* body mass index, *BP* blood pressure, *bpm*, beats per minute, *NYHA* New York Heart Association, *CAD* coronary artery disease, *PM* pace maker, *ICD* intracardiac defibrillator, *AT II,* angiotensin II, *ACE* angiotensin converting enzyme, *LA* left atrium, *RA* right atrium, *LVEDD* left ventricular end-diastolic diameter, *LVEF* left ventricular ejection fraction, *IVS* intraventricular septum, *RVEDD* right ventricular end-diastolic diameter, *RV-TDI* right ventricular tissue Doppler index, *TAPSE* tricuspid annular plane systolic excursion, *RV-GLS* right ventricular global longitudinal strain, *TR* tricuspid regurgitation, *sPAP* systolic pulmonary artery pressure, *IVC* inferior vena cava, *NT-pro BNP* N-terminal pro brain natriuretic peptide, *eGFR* estimated glomerular filtration rate, *ASAT* aspartate aminotransferase, *ALAT* alanine aminotransferase, *GGT* gamma glutamyltransferase, *LDH* lactate dehydrogenase, *CRP* C-reactive protein

*Indexed to body surface area

**Log values were used for analysis

## Discussion

Our data show that the presence of pleural effusion in patients with AL is associated with poor RV function and higher serum levels of NT-pro BNP and troponin T. While cardiac biomarkers were also higher in patients with ATTR and pleural effusion, there was no difference in RV function depending on the presence or absence of pleural effusion in these patients. We found that patients with AL presenting with pleural effusions had poor clinical outcomes and that concomitant or isolated pericardial effusions were not associated with worse outcomes in these patients.

Few previous studies have described the effects of effusions on outcome and even less considered both pericardial and pleural effusions in CA. Berk et al. previously proposed that pleural effusion may not be an effect of right heart failure, as their data showed no correlation of right heart parameters and presence of pleural effusions in their pure AL cohort [14]. In contrast, we did see a relationship between echocardiographically measured right heart parameters and pleural effusions in patients with AL. The reason for these discordances may be better image quality almost 16 years later and development of more sensitive imaging parameters such as 2D-strain imaging, which were not measured in the mentioned study and could potentially detect more subtle forms of RV dysfunction [23].

Interestingly, our data showed that the association between pleural effusion and RV function was only present in AL, but not in ATTR. However, patients with ATTR and pleural effusions had significantly lower serum albumin levels compared to patients without pleural effusions (Table 3). This finding suggests that pleural effusions may develop as a result of decreased plasma oncotic pressure in patients with ATTR, rather than from RV failure and could point to a difference in pathophysiologic mechanisms of effusion in different subtypes of CA.

Based on our data, as well as previous studies it can be hypothesized that pleural and pericardial effusions may not simply be explained by the failure of the RV. Effusions could also be a result of local amyloid infiltration of the serosae as well as tissue inflammation. In addition, hypalbuminemia could play a role in the development of pleural effusions in addition to elevated filling pressures [24].

Deeper knowledge of the pathophysiology and prognostic relevance of effusions in CA is important to guide physicians in their decision whether or not to drain effusions in these patients. While pericardial effusions in our patient cohort were generally small and hemodynamically not relevant, the question of pericardiocentesis is probably not as central as the decision for pleurocentesis in clinical practice. An interesting question which our data do not answer is, if drainage of pleural fluid results in symptom control or change in cardiac or overall prognosis. Since pericardial effusions could only be detected by ultrasound in some cases, we emphasize the importance of assessment of pleural effusion as part of a standard echocardiography exam, as effusions may be too small to detect in chest X-ray.

### Limitations

We are aware of the limitations, which are associated with the single-center design of the present study. However, this also implicates advantages regarding the consistency of diagnostic work-up, treatment and follow-up. Infiltration of pericardial or pleural tissue can of course not be certified without histological tissue characterization, which is not feasible in pre-mortem patient cohorts for obvious reasons. Even contrast CMR imaging would not be able to detect, let alone quantify amyloid deposits in the pericardium due to technical limitations. Effusion samples were not collected, because most patients did not undergo thoracocentesis, but were medically treated. Analysis of effusions could have given more insight into their underlying cause. Furthermore, we acknowledge, that diuretic treatment may have influenced the results of this study.

## Conclusion

Pericardial and pleural effusions are present in more then 50% of patients with CA and assessment of effusion should be included in standard echocardiographic examination of all patients with suspected or definite diagnosis of CA. Pleural-, but not pericardial effusions were associated with adverse cardiac outcomes. The development of effusions in these patients may not simply be a sign of right heart failure, but may have a multifactorial genesis including decrease in serum albumin levels resulting in changes of oncotic pressures or local inflammation due do amyloid infiltration of the myocardium or pleural and pericardial serosae.

## Electronic supplementary material

Below is the link to the electronic supplementary material.

Supplementary file1 (DOCX 19 kb)
