# Supplementary material for: Prognostic implications of pericardial and pleural effusion in patients with cardiac amyloidosis
Source: Clin Res Cardiol. 2020 Sep 10;110(4):532–43. doi: 10.1007/s00392-020-01698-7 (PMC8055634; doi:10.1007/s00392-020-01698-7)
Supplement: Supplementary file 1 — Supplementary file1 (DOCX 19 kb) [file 392_2020_1698_MOESM1_ESM.docx]

**Table S1-** Baseline echocardiographic and laboratory parameters of patients with light chain amyloidosis (n=58) with and without pericardial effusion*.

|  | **No pericardial effusion** (n=37) | **Pericardial effusion** (n=21) |  |
| --- | --- | --- | --- |
| ***Echocardiography parameters*** | |  |  |
| RV diameter, mm | 36.0 (28.0-39.0) | 32.0 (28.0-34.0) | 0.095 |
| RA length, mm | 59.0 (53.0-65.0) | 56.0 (49.0-60.0) | 0.094 |
| **RV strain basal**, -% | 16.0 (21.0-13.0) | 13.0 (17.0-8.5) | **0.028** |
| **RV strain mid**, -% | 18.0 (21.0-14.0) | 14.0 (18.5-9.5) | **0.024** |
| RV strain apical, -% | 18.0 (21.0-13.0) | 15.5 (18.5-10.5) | 0.087 |
| RV- GLS, -% | 17.7 (20.7-13.7) | 14.5 (18.7-9.7) | 0.058 |
| RV- TDI, m/s | 0.13 (0.10-0.16) | 0.12 (0.10-0.15) | 0.323 |
| TAPSE, mm | 16.0 (14.0-18.0) | 15.0 (12.0-22.0) | 0.829 |
| TR velocity, m/s | 2.8 (2.5-3.3) | 3.0 (2.8-3.6) | 0.091 |
| sPAP, mmHg | 46.5 (34.5-59.5) | 41.0 (39.0-61.0) | 0.899 |
| IVC diameter, mm | 20.5 (18.0-25.0) | 18.0 (15.5-22.5) | 0.304 |
| ***Laboratory parameters*** |  |  |  |
| NT-pro BNP, pg/mL | 3592.0 (1783.0-10171.0) | 4870.0 (3385.0-9674.0) | 0.605 |
| Troponin T, ng/L | 54.0 (22.0-126.0) | 66.0 (38.0-231.0) | 0.465 |
| Gamma GT, U/L | 49.0 (27.0-128.0) | 76.0 (46.0-100.0) | 0.502 |
| ASAT, U/L | 23.0 (19.0-29.0) | 28.0 (20.0-35.0) | 0.118 |
| **ALAT**, U/L | 18.0 (15.0-26.0) | 25.0 (18.0-34.0) | **0.027** |
| Albumin, U/L | 33.9 (27.8-40.8) | 39.9 (35.4-41.9) | 0.139 |
| CRP, mg/dl | 0.5 (0.1-2.3) | 0.4 (0.1-1.0) | 0.298 |
| eGFR, mL/min/1.73m^2^ | 41.8 (23.3-55.9) | 51.4 (40.4-60.2) | 0.180 |

* 12 patients of the shown population had additional pericardial effusion.

Continuous variables are given in mean and interquartile range.

RV indicates right ventricle; RA, right atrium; RV-GLS, right ventricular global longitudinal strain; TDI, tissue Doppler index; TAPSE, tricuspid annular plane systolic excursion; TR, tricuspid regurgitation; sPAP, systolic pulmonary artery pressure; IVC, inferior vena cave; NT-pro BNP, N-terminal pro brain natriuretic peptide; Gamma GT, gamma glutamyltransferase; ASAT, aspartate aminotransferase; ALAT; alanine aminotransferase; CRP, C-reactive protein and eGFR, estimated glomerular filtration rate calculated by the modification of diet in renal disease (MDRD) formula.

**Table S2-** Baseline echocardiographic and laboratory parameters of patients with transthyretin amyloidosis (n=85) with and without pericardial effusion*.

|  | | **No pericardial effusion** (n=63) | **Pericardial effusion** (n=22) |  |
| --- | --- | --- | --- | --- |
| ***Echocardiography parameters*** | | |  |  |
| RV diameter, mm | 33.0 (29.0-38.0) | | 35.0 (31.0-42.0) | 0.401 |
| **RA length**, mm | 59.0 (53.0-63.0) | | 66.0 (60.0-72.0) | **0.003** |
| RV strain basal, -% | 14.0 (21.0-11.0) | | 15.0 (20.0-10.0) | 0.828 |
| RV strain mid, -% | 15.0 (21.0-11.0) | | 18.0 (19.0-10.0) | 0.960 |
| RV strain apical, -% | 17.0 (21.0-11.0) | | 16.0 (20.0-11.0) | 0.475 |
| RV- GLS, -% | 14.3 (21.0-11.3) | | 17.3 (19.0-10.0) | 0.823 |
| RV- TDI, m/s | 0.10 (0.09-0.12) | | 0.10 (0.07-0.13) | 0.645 |
| TAPSE, mm | 17.0 (13.0-19.0) | | 15.0 (11.0-19.0) | 0.406 |
| TR velocity, m/s | 2.9 (2.6-3.2) | | 2.8 (2.7-3.3) | 0.606 |
| sPAP, mmHg | 43.0 (35.0-55.0) | | 41.0 (37.0-58.0) | 0.918 |
| **IVC diameter**, mm | 21.0 (17.0-23.0) | | 21.0 (18.0-28.5) | 0.230 |
| ***Laboratory parameters*** |  | |  |  |
| **NT-pro BNP**, pg/mL | 1918.0 (999.5.0-3973.0) | | 3514.5 (1825.0-6714.0) | **0.049** |
| **Troponin T**, ng/L | 39.0 (28.0-68.0) | | 65.0 (40.0-97.0) | **0.043** |
| Gamma GT, U/L | 67.0 (32.0-135.0) | | 78.5 (47.0-140.0) | 0.290 |
| ASAT, U/L | 30.0 (24.0-35.0) | | 29.0 (24.0-35.0) | 0.972 |
| ALAT, U/L | 24.0 (18.0-32.0) | | 27.0 (22.0-36.0) | 0.197 |
| Albumin, U/L | 43.3 (38.7-45.7) | | 40.4 (36.5-43.3) | 0.126 |
| CRP, mg/dl | 0.2 (0.1-1.0) | | 0.2 (0.1-0.6) | 0.916 |
| eGFR, mL/min/1.73m^2^ | 53.5 (43.1-70.7) | | 56.3 (43.2-88.9) | 0.609 |

* 12 patients of the shown population had additional pericardial effusion.

Continuous variables are given in mean and interquartile range.

RV indicates right ventricle; RA, right atrium; RV-GLS, right ventricular global longitudinal strain; TDI, tissue Doppler index; TAPSE, tricuspid annular plane systolic excursion; TR, tricuspid regurgitation; sPAP, systolic pulmonary artery pressure; IVC, inferior vena cave; NT-pro BNP, N-terminal pro brain natriuretic peptide; Gamma GT, gamma glutamyltransferase; ASAT, aspartate aminotransferase; ALAT; alanine aminotransferase; CRP, C-reactive protein and eGFR, estimated glomerular filtration rate calculated by the modification of diet in renal disease (MDRD) formula.
